# A novel prognostic signature of metastasis-associated genes and personalized therapeutic strategy for lung adenocarcinoma patients

**DOI:** 10.18632/aging.204169

**Published:** 2022-07-12

**Authors:** Zhihao Wang, Yusi Liu, Xiaoqian Zhan, Xi Wang, Chao Zhang, Lingzhi Qin, Liwei Liu, Shenghui Qin

**Affiliations:** 1 Institute of Pathology, Tongji Hospital, Tongji Medical College, Huazhong University of Science and Technology, Wuhan 430030, China; 2 Hubei University of Science and Technology, Xianning Medical College, Xianning 437100, China

**Keywords:** lung adenocarcinoma, metastasis, prognostic, signature, candidate drugs

## Abstract

Lung adenocarcinoma (LUAD) is a highly invasive and metastatic malignant tumor with high morbidity and mortality. This study aimed to construct a prognostic signature for LUAD patients based on metastasis-associated genes (MAGs). RNA expression profiles were downloaded from the Cancer Genome Atlas (TCGA) database. RRA method was applied to identify differentially expressed MAGs. A total of 192 significantly robust MAGs were determined among seven GEO datasets. MAGs were initially selected through the Lasso Cox regression analysis and 6 MAGs were included to construct a prognostic signature model. Transcriptome profile, patient prognosis, correlation between the risk score and clinicopathological features, immune cell infiltration characteristics, immunotherapy sensitivity and chemotherapy sensitivity differed between low- and high-risk groups after grouping according to median risk score. The reliability and applicability of the signature were further validated in the GSE31210, GSE50081 and GSE68465 cohort. CMap predicted 62 small molecule drugs on the base of the prognostic MAGs. Targeted drug staurosporine had hydrogen bonding with Gln-172 of SLC2A1, which is one of MAGs. Staurosporine could inhibit cell migration in A549 and H1299. We further verified mRNA and protein expression of 6 MAGs in A549 and H1299. The signature can serve as a promising prognostic tool and may provide a novel personalized therapeutic strategy for LUAD patients.

## INTRODUCTION

Lung adenocarcinoma (LUAD) is the most common type of lung cancer, which has the highest morbidity and mortality in China and even the world. Current medical advances in the treatment of LUAD, including surgery, radiotherapy, chemotherapy, and systemic therapy, have greatly improved patient survival [1, 2]. More and more LUAD genomic studies have characterized important targeted therapeutic [3], such as EGFR [4], ALK [5] and c-MET [6]. Moreover, more and more LUAD patients are also benefiting from PDL1 immunotherapy [7]. Despite these advances in clinical treatment of LUAD, the prognosis of advanced lung adenocarcinoma is still poor, and most patients die of diagnosis at advanced stage and with distant metastasis.

Although two studies have previously been conducted by our team to identify numerous biomarkers associated with the survival of LUAD for predicting prognosis, including metabolism-related genes [8] and epigenetic-related prognostic signature [9]. However, since most patients with advanced lung adenocarcinoma die of tumor recurrence and metastasis, it is particularly important to analyze the predictive genes related to recurrence and metastasis. In recent years, more and more articles have been reported about the prognosis of metastasis-related genes, such as breast cancer [10], colon adenocarcinoma [11], glioma [12]. Qing Cao.et. al have recently reported 6 metastasis-associated six lncRNA signature that had the greatest prognostic value for lung cancer [13]. However, the most common pathological type of metastasis lung cancer is lung adenocarcinoma, which is rising in incidence and mortality in recent years.

In this study, the differential mRNA expression data of LUAD from the HCMDB and GEO databases were analyzed to identify key genes. The integrated bioinformatics analysis by investigating the functions and pathways of the gene was used to further investigated their potentiality of being biomarkers in LUAD. A metastasis-associated prognostic signature based on six MAGs was constructed by Lasso and multiple Cox regression analyses. The prognostic value of the genes was evaluated using the ROC curve (Receiver Operating Characteristic Curve) and survival analysis. The reliability and applicability of the signature were further validated in the GSE31210, GSE50081 and GSE68465 cohort. Besides, correlation between the risk score and clinicopathological features, immune microenvironment characteristics, immunotherapy sensitivity, chemotherapy sensitivity and candidate drugs targeting the risk signature were analyzed in LUAD patients. *In vitro* experiment was conducted to confirm the mRNA and protein expression 6 MAGs in LUAD cell lines. Effect of candidate drug staurosporine on cell metastasis was conducted by migration experiment.

## RESULTS

### Identification of differentially expressed MAGs

**Figure 1:**
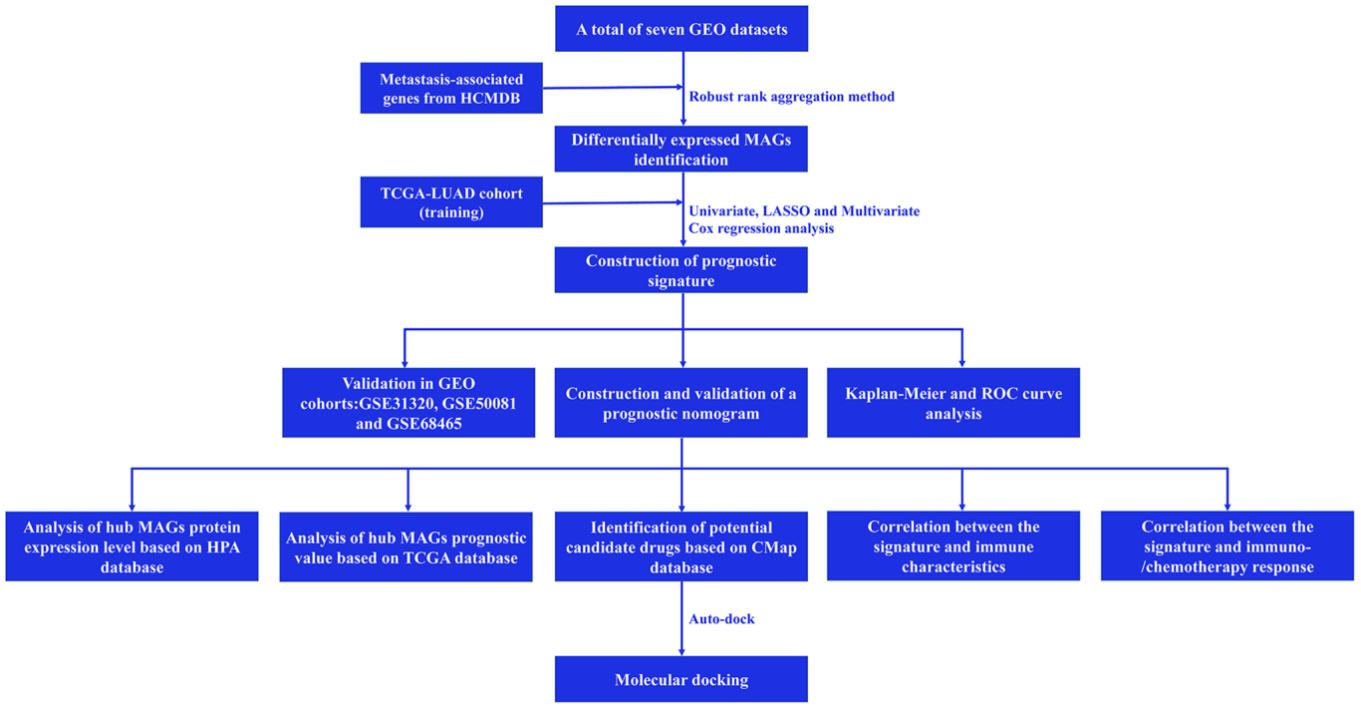
Flow chart of this study.

**Figure 2:**
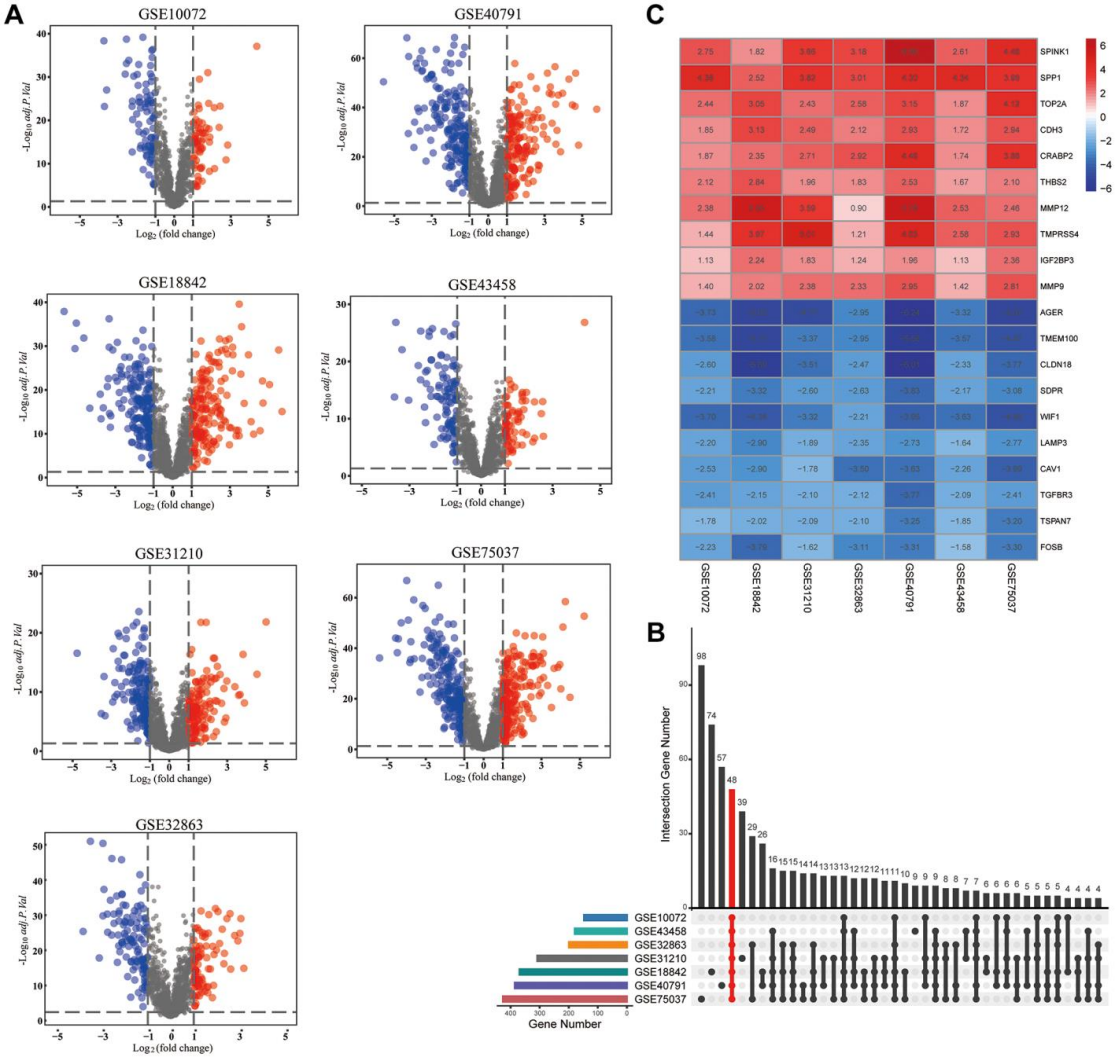
**Identification of differentially expressed metastasis-associated genes (MAGs).** (**A**) The volcano plots of differentially expressed MAGs in each GEO cohort, (**B**) Intersection plot of the MAGs in these cohorts, (**C**) The expression heatmap of the top 20 differentially expressed MAGs.

A simplified protocol flow chart of this study was presented in Figure 1. 1938 MAGs were obtained from the HCMDB database. Seven GEO datasets were used to screen differentially expressed MAGs (Table 1). Based on the cutoff criteria as before, 147 differentially expressed MAGs (84 downregulated and 63 upregulated MAGs) were identified in GSE10072 dataset, 369 differentially expressed MAGs (193 downregulated and 176 upregulated MAGs) were identified in GSE18842 dataset, 308 differentially expressed MAGs (172 downregulated and 136 upregulated MAGs) were identified in GSE31210 dataset, 199 differentially expressed MAGs (113 downregulated and 86 upregulated MAGs) were identified in GSE32863 dataset, 199 differentially expressed MAGs (113 downregulated and 86 upregulated MAGs) were identified in GSE40791 dataset, 179 differentially expressed MAGs (109 downregulated and 70 upregulated MAGs) were identified in GSE43458 dataset, 426 differentially expressed MAGs (220 downregulated and 206 upregulated MAGs) were identified in GSE75037 dataset (Figure 2A). Due to only 48 common MAGs were found between the seven GEO datasets (Figure 2B), RRA method was used to identify MAGs and finally 192 significantly robust MAGs were determined, including 109 downregulated and 83 upregulated MAGs (Figure 2C).

**Table 1:** Characteristics of GEO datasets included in the study.

**GEO ID**	**Tissue**	**Platform ID**	**Number of samples**	
			**Normal**	**Tumor**
GSE10072	Lung	GPL96	49	58
GSE18842	Lung	GPL570	45	46
GSE31210	Lung	GPL570	20	226
GSE32863	Lung	GPL6884	58	58
GSE40791	Lung	GPL570	100	94
GSE43458	Lung	GPL6244	30	80
GSE75037	Lung	GPL6884	83	83

Abbreviations: GPL: Gene Expression Omnibus Platform; GSE: Gene Expression Omnibus Series.

### Functional enrichment analysis

**Figure 3:**
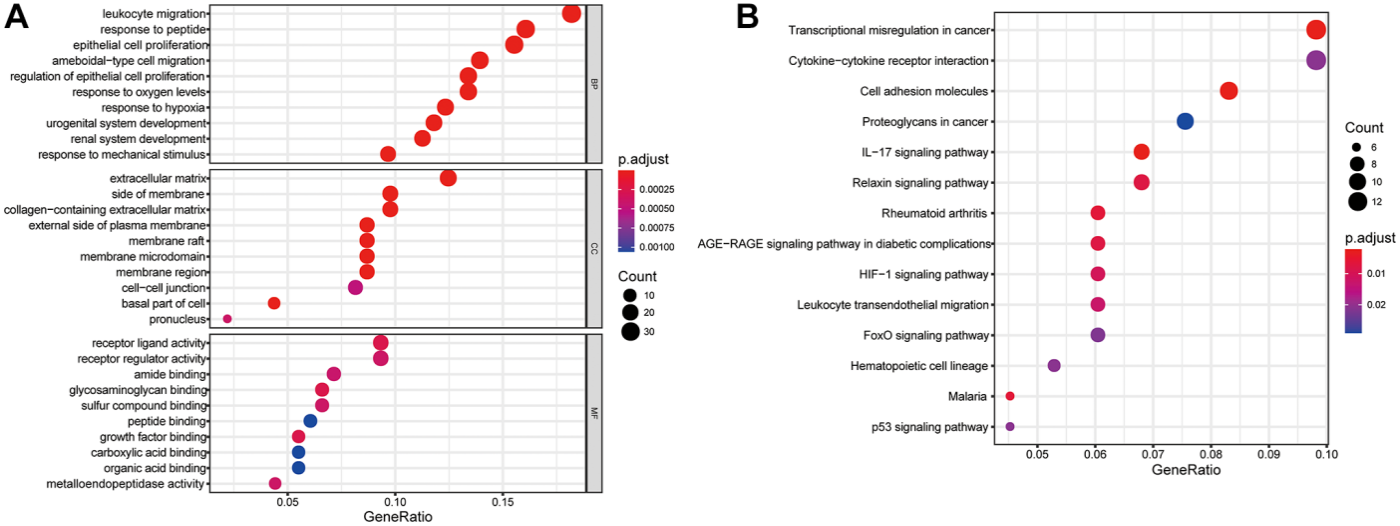
**Gene functional enrichment analysis of differentially expressed MAGs.** (**A**) GO and (**B**) KEGG analyses.

GO analysis were carried out to investigate the potential biological function of the 192 MAGs and KEGG pathway enrichment analyses were carried out to found the promising signaling pathways. We found that 192 MAGs were mainly enriched in leukocyte migration, response to peptide, epithelial cell proliferation, extracellular matrix, receptor regulator activity and et al. (Figure 3A). Also, these MAGs were mainly enriched in transcriptional misregulation in cancer, proteoglycans in cancer, IL-17 signaling pathway and et al. (Figure 3B).

### Construction of metastasis-associated prognostic signature

**Figure 4:**
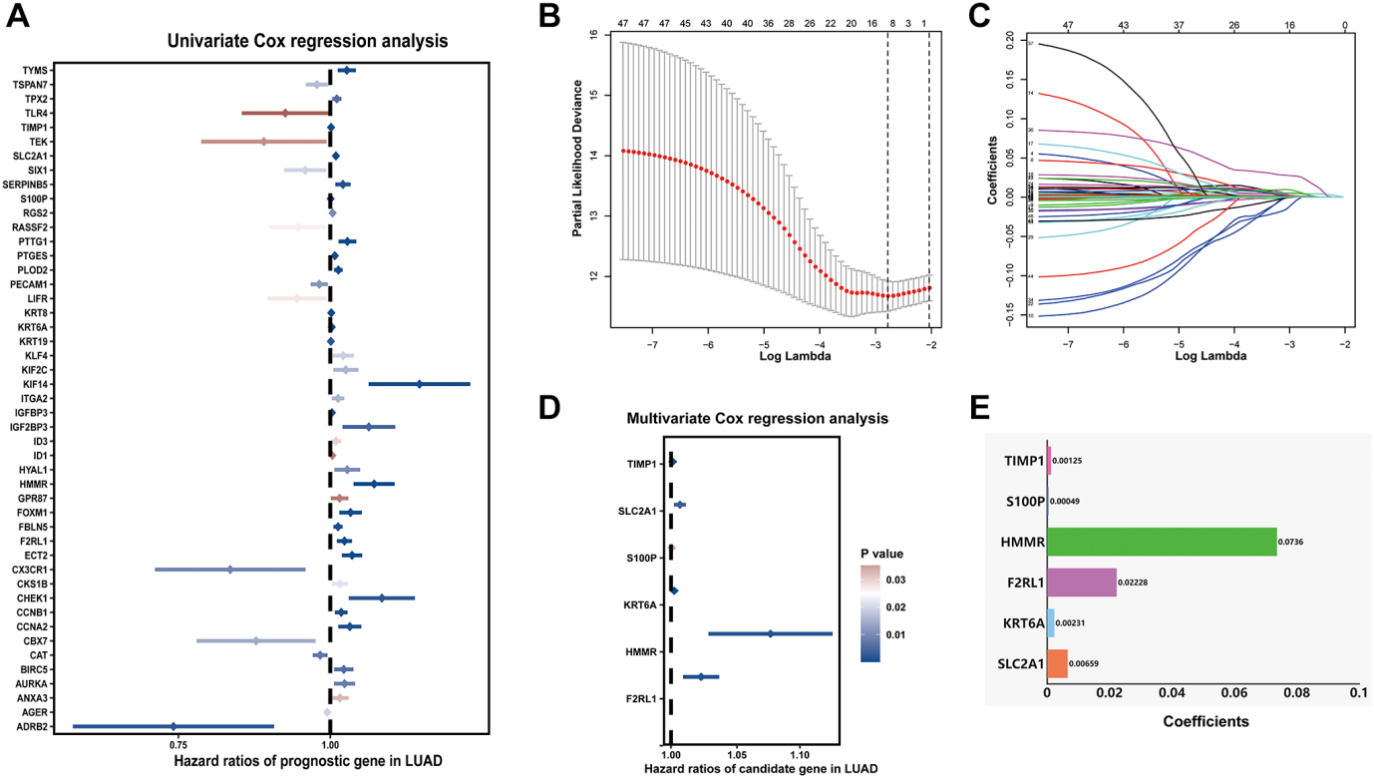
**Prognostic signature for LUAD patients based on differentially expressed MAGs.** (**A**) Univariate Cox regression analysis showed that theses MAGs significantly correlated with clinical prognosis, (**B**) Partial likelihood deviance for the Lasso regression, (**C**) Lasso regression analysis, (**D**) Multivariate Cox regression analysis revealed six independent prognostic MAGs, (**E**) Coefficients of these genes.

**Figure 5:**
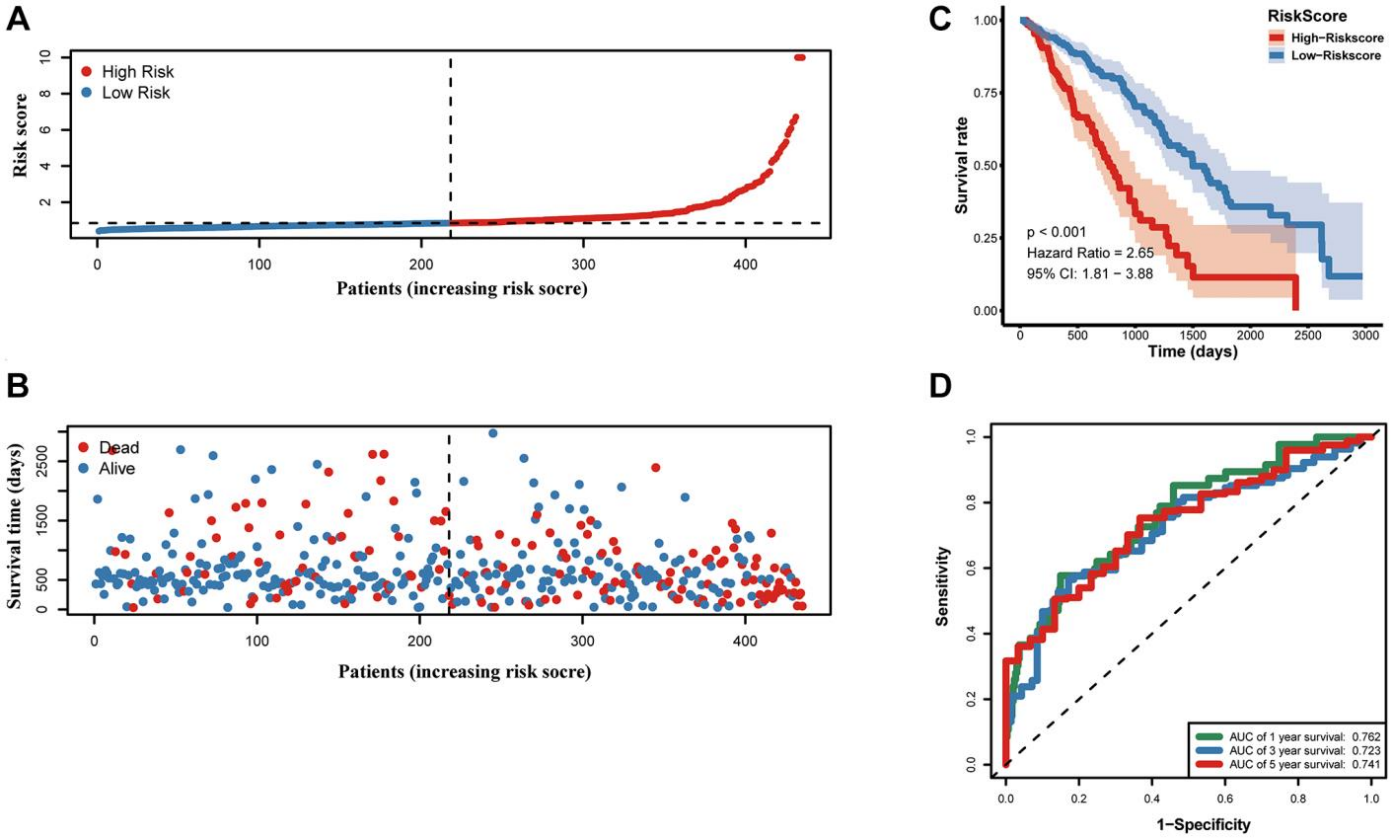
**Prognostic value of the prognostic signature in the TCGA cohort.** (**A**) the risk score and (**B**) the survival status of LUAD patients, (**C**) Kaplan-Meier survival analysis, (**D**) ROC analysis.

Samples from TCGA-LUAD were classified as the training cohort. Univariate cox regression analysis was conducted on 192 differentially expressed MAGs. A total of 47 genes associated with prognosis were identified with adjusted *P* value < 0.05 (Figure 4A). After Lasso and multiple Cox regression analyses, a metastasis-associated prognostic signature based on six MAGs (TIMP1, S100P, HMMR, F2RL1, KRT6A, and SLC2A1) was constructed (Figure 4B–4D). The coefficients of these genes were displayed in Figure 4E, and the signature risk score was defined as the sum of the expression levels of the coefficients-weighted genes. LUAD patients were stratified into the high- and low-risk groups by the mean risk score. The risk score, survival status and survival time of patients were respectively shown in Figure 5A**–**5C. Low-risk group patients have significantly better OS than high-risk group, indicating that the risk score had a prognostic value (Figure 5C). The AUCs for 1-, 3-, 5-year OS rate were 0.762, 0.723, and 0.741, respectively (Figure 5D, *P* < 0.001).

### Correlation between the risk score and clinicopathological features

**Figure 6:**
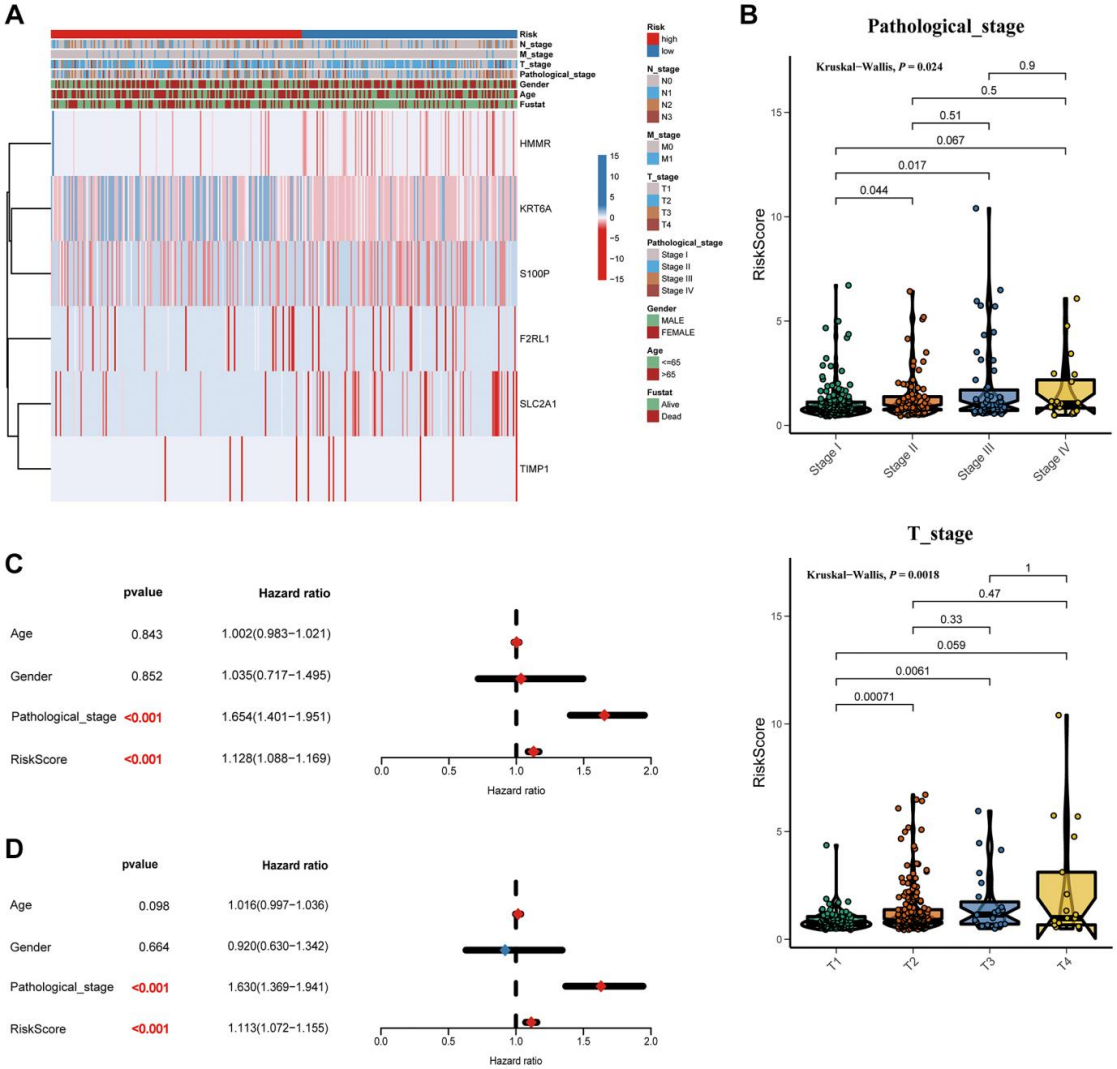
**Correlation between the risk score and clinicopathological features.** (**A**) Heatmap and clinicopathological features of patients classified by signature. (**B**) Boxplots showed the risk score with different pathological_stage and T_stage. Univariate (**C**) and multivariate (**D**) Cox regression analyses.

**Figure 7:**
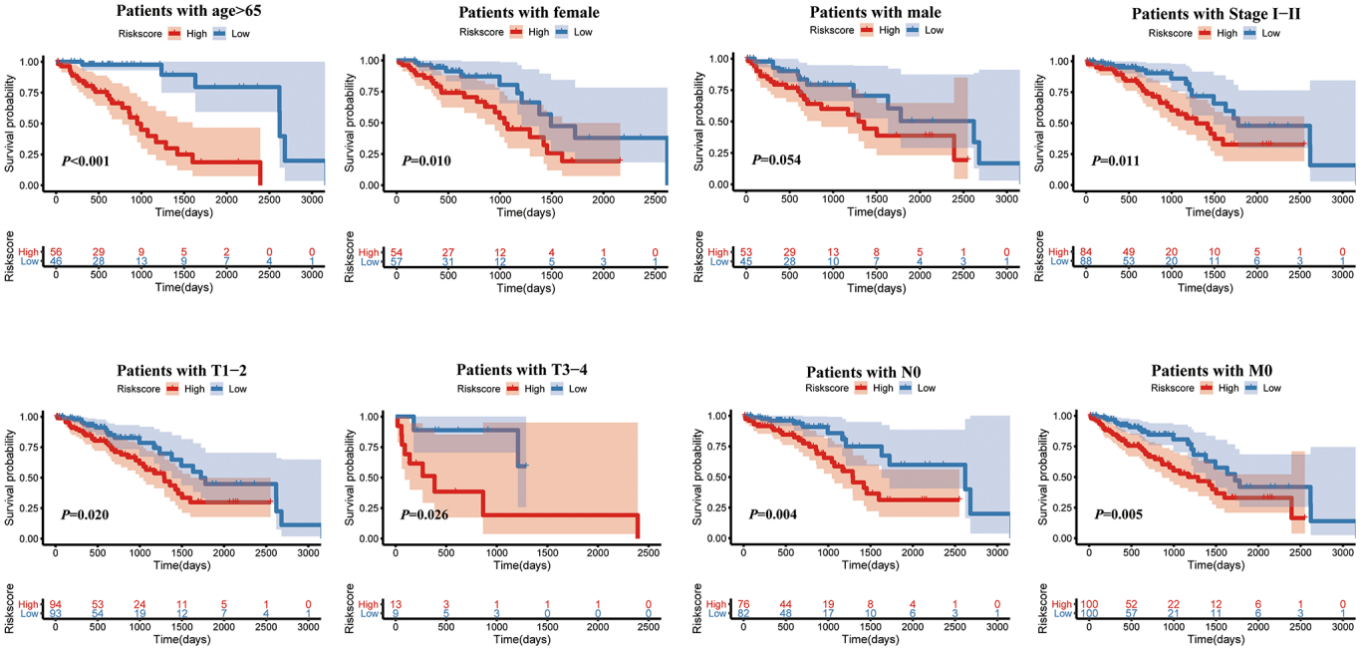
Kaplan-Meier survival analysis of the risk score for LUAD patients grouped by clinicopathological features.

Furthermore, as shown in the heatmap, the expression levels of TIMP1, S100P, HMMR, F2RL1, KRT6A, and SLC2A1 were increased in high risk score group, (Figure 6A). The higher pathological stage was concomitant with a higher risk score. Moreover, patients with higher risk factor scores had higher T-stages (Figure 6B). Univariate (Figure 6D) and multivariate (Figure 6C) Cox analyses showed that both the risk score and pathological stage were independent risk factors. The prognostic value of the signature was analyzed via stratification analysis. We also combined the signature model with clinical risk factors (including age, sex, clinical stage, and TNM stage) to better exploit its prognostic predictive efficiency in lung adenocarcinoma patients, and the results showed that when patients were exposed to the same clinical risk factors (such as age >65, Female, Stage I–II, T-stage (T1/2, or T3/4), N-stage (N0), and M-stage (M0)), the high risk group had a significantly worse prognosis (Figure 7).

### Validation of metastasis-associated prognostic signature

**Figure 8:**
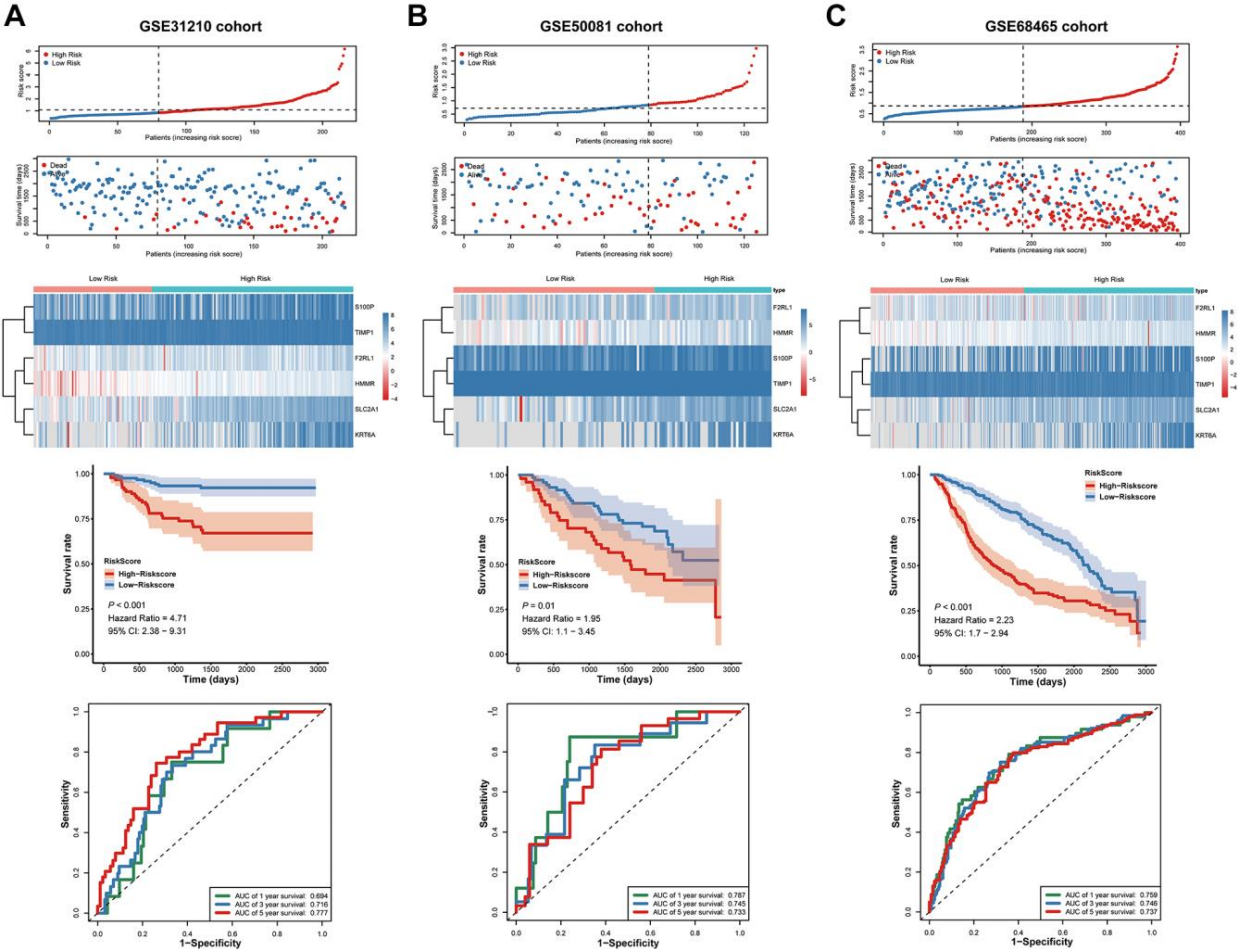
**Validation of the prognostic signature in the GSE31210, GSE50081 and GSE68465 cohort.** (**A**–**C**) the risk score and the survival status of LUAD patients, Kaplan-Meier survival analysis, ROC analysis.

For validating whether the signature showed robust prognostic value, we also validated the metastasis-associated prognostic signature in other three independent cohorts (GSE31210, GSE50081, and GSE68465 cohort). In line with results in TCGA cohort, patients with high-risk scores exhibited significantly poorer OS relative to those with low-risk scores (Figure 8A–8C).

Next, we used ROC Curves to test the accurately of the prognostic signature for predicting patients' 1-, 3-, and 5-year OS. The analysis results showed that AUC values in all three independent cohorts have statistically significant (Figure 8A–8C). These results suggested that the signature was capable of general application and had a robust performance in predicting LUAD patients’ prognosis.

### Construction of a nomogram based on the prognostic signature

**Figure 9:**
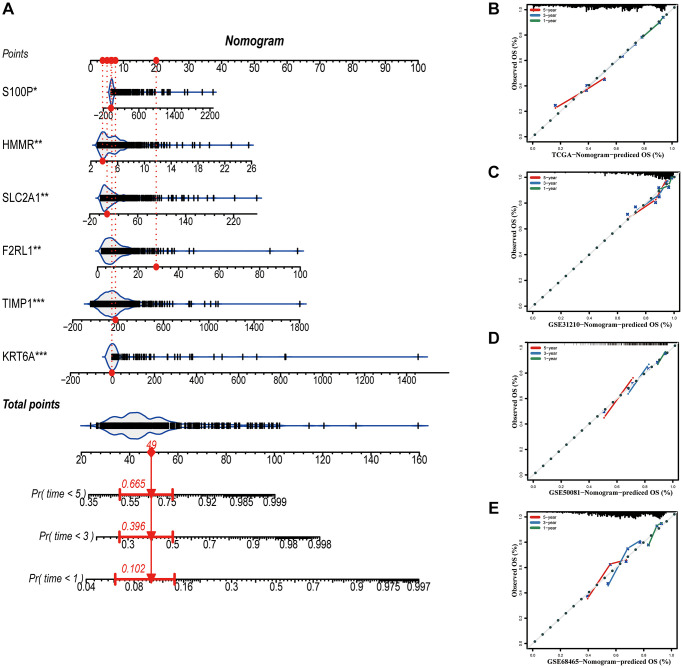
**Construction of a nomogram based on the prognostic signature.** (**A**) The nomogram based on the signature. (**B**–**E**) Calibration curves of nomogram for the signature. ^*^P < 0.05; ^**^P < 0.01; ^***^P < 0.001.

**Figure 10:**
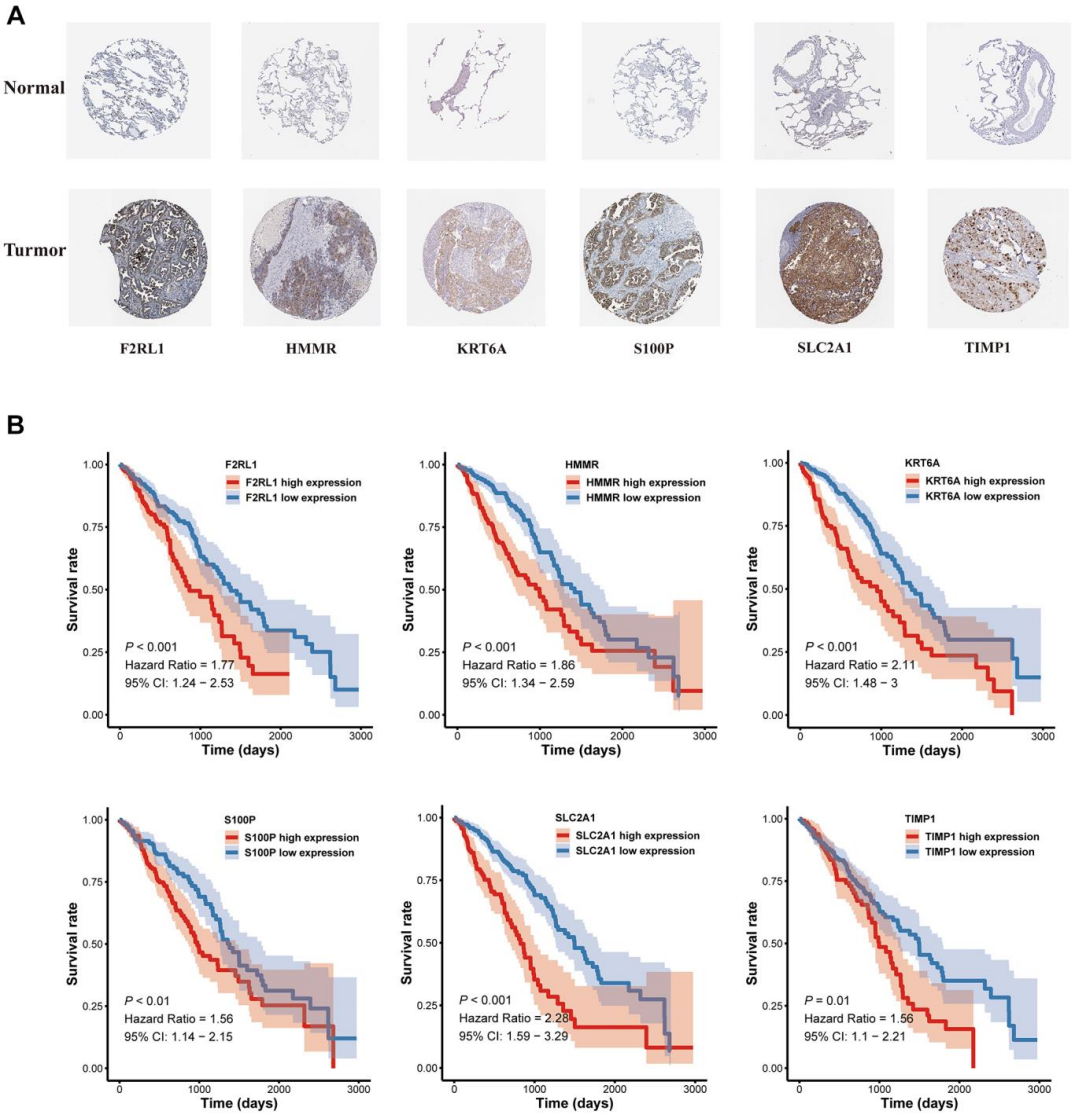
**The expression and survival analyses of the six MAGs.** (**A**) The representative protein expression of MAGs in lung adenocarcinoma tissue and normal tissue. (**B**) Kaplan-Meier survival analysis.

A nomogram based on metastasis-associated prognostic signature was constructed to provide clinicians a quantitative method, which could individually predict 1-, 3- and 5-year OS of each LUAD patients (Figure 9A). The calibration curve of the nomogram demonstrated good consistency with the predictions for 1-, 3- and 5-year OS in four cohorts (Figure 9B–9E). In addition, results from the HPA database showed that protein expression of F2RL1, HMMR, KRT6A, S100P, and SLC2A1 were significantly increased in LUAD tissues (Figure 10A). The prognostic value of 6 genes was analyzed using TCGA-LUAD dataset, the results illustrated that high expression of these genes suggested a worse prognosis for patients (Figure 10B).

### Characteristics of immune microenvironment and immunotherapy sensitivity in two risk factor groups

**Figure 11:**
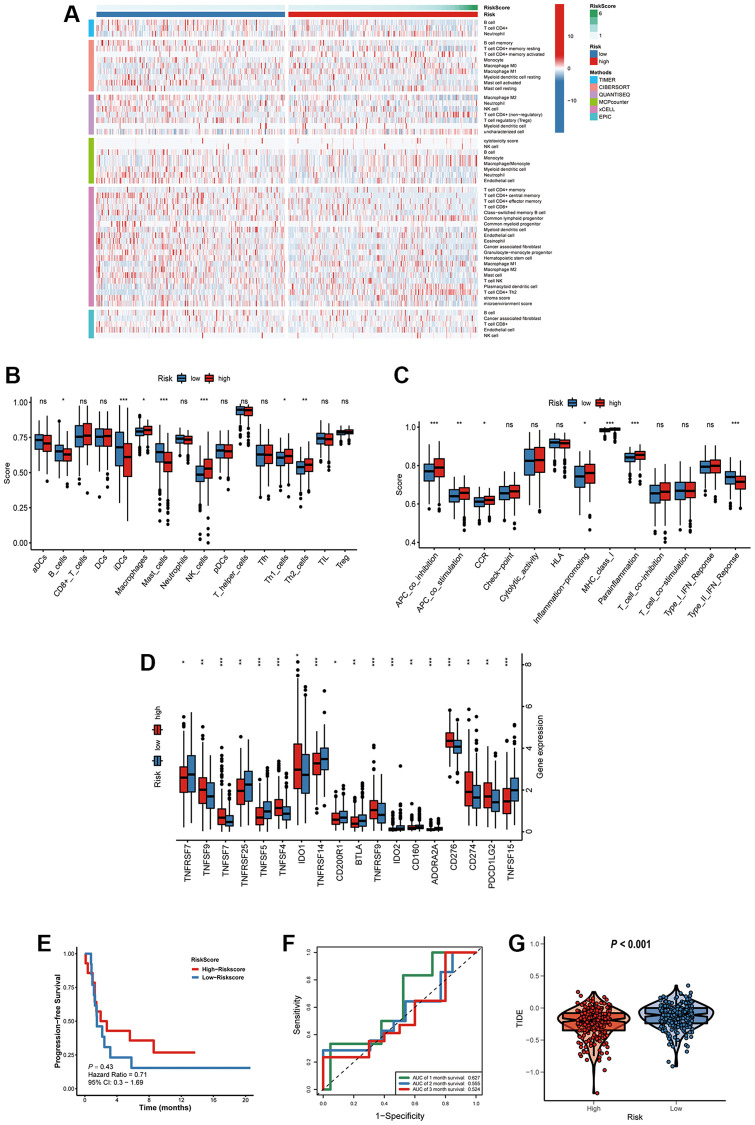
**Characteristics of immune microenvironment and immunotherapy sensitivity in two risk factor groups.** (**A**) The heatmap of immune responses. (**B**) The scores of immune cells and (**C**) immune-related functions in high and low risk groups. (**D**) The expression of immune checkpoints among high and low risk groups. (**E**) Kaplan-Meier survival curve of GSE135222 cohort for PFS. (**F**) ROC analysis in the GSE135222. (**G**) Compared the score of TIDE between high and low risk groups. ^*^P < 0.05; ^**^P < 0.01; ^***^P < 0.001; ns, not significant.

The immune responses between high- and low-risk groups were evaluated by using TIMER, CIBERSORT, QUANTISEQ, MCPcounter, xCELL and EPIC algorithms (Figure 11A). Single-sample gene set enrichment analysis (ssGSEA) was applied to quantify the infiltrating score of tumor-infiltrating immune cells and immune-related pathways between the two groups, as shown in Figure 11B, the high-risk group exhibited higher levels of infiltration of immune cells, especially of macrophages, natural killer (NK) cells, T helper (Th) cells (Th1, and Th2 cells). Moreover, high-risk group patients showed higher scores of antigen presenting cell (APC)-co-inhibition, APC-co- stimulation, chemokine receptor (CCR), inflammation-promoting, and parainflammation, the lower score of type II IFN response (Figure 11C). Immune checkpoint blockade therapy has become an effective strategy for the treatment of LUAD patients [14]. Therefore, the expression levels of immune checkpoints between the two groups were explored, results demonstrated that high-risk group patients showed higher expression of CD274 (PD-L1), PDCD1LG2 (PD-L2), TNFSF4/7/9, TNFRSF9 and IDO1(Figure 11D).

To verify the prognostic value of the signature for immunotherapy sensitivity, GSE135222 dataset from LUAD patients with immunotherapy was selected. Based on the signature formulate, the risk score of each patient in the GSE135222 cohort was calculated. The high risk group undergoing anti-PD-1/PD-L1 therapy had a better progression-free survival (PFS) than low risk group, implying that the signature reflects sensitivity to immunotherapy (Figure 11E), and the AUC value for predicting the 3-month PFS was 0.627 (Figure 11F). As shown in Figure 11G, high-risk group had a lower TIDE score, suggesting that these patients might have a higher efficacy and better outcome after receiving the immunotherapy. (Figure 11G).

### Chemotherapy sensitivity and candidate drugs targeting the risk signature

**Figure 12:**
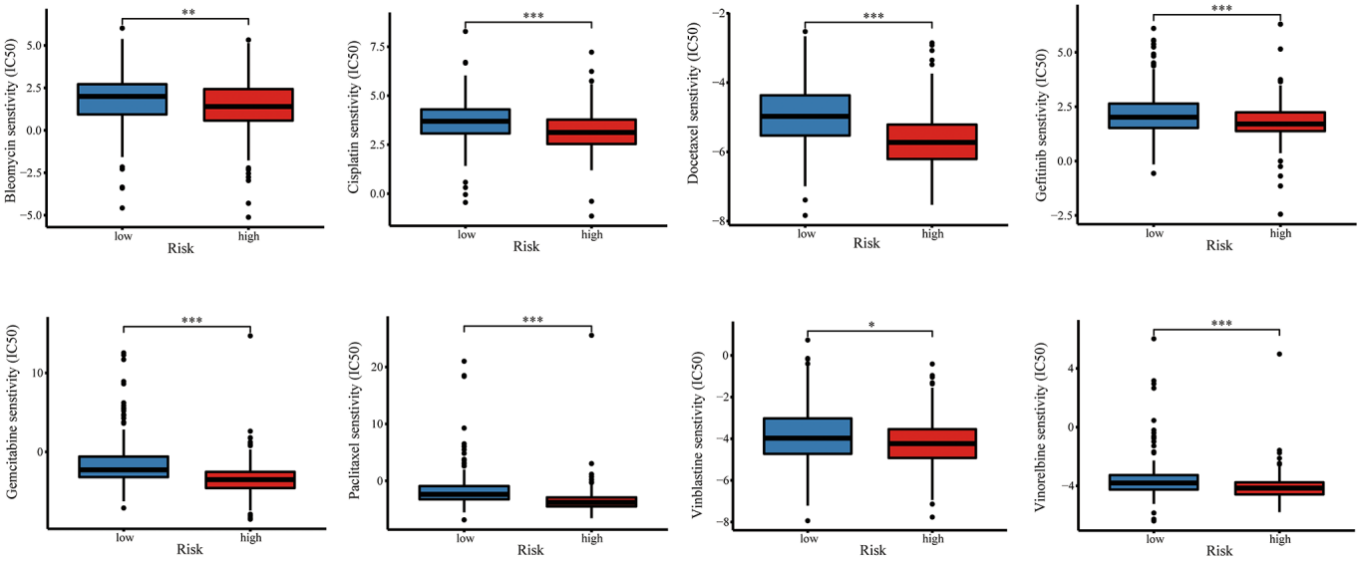
**The relationship of risk signature with chemotherapy response.** Difference of IC50 value between high- and low-risk groups for common chemotherapeutics drugs including Bleomycin, Cisplatin, Docetaxel, Gefitinib, Gemcitabine, Paclitaxel, Vinblastine, and Vinorelbine.

**Figure 13:**
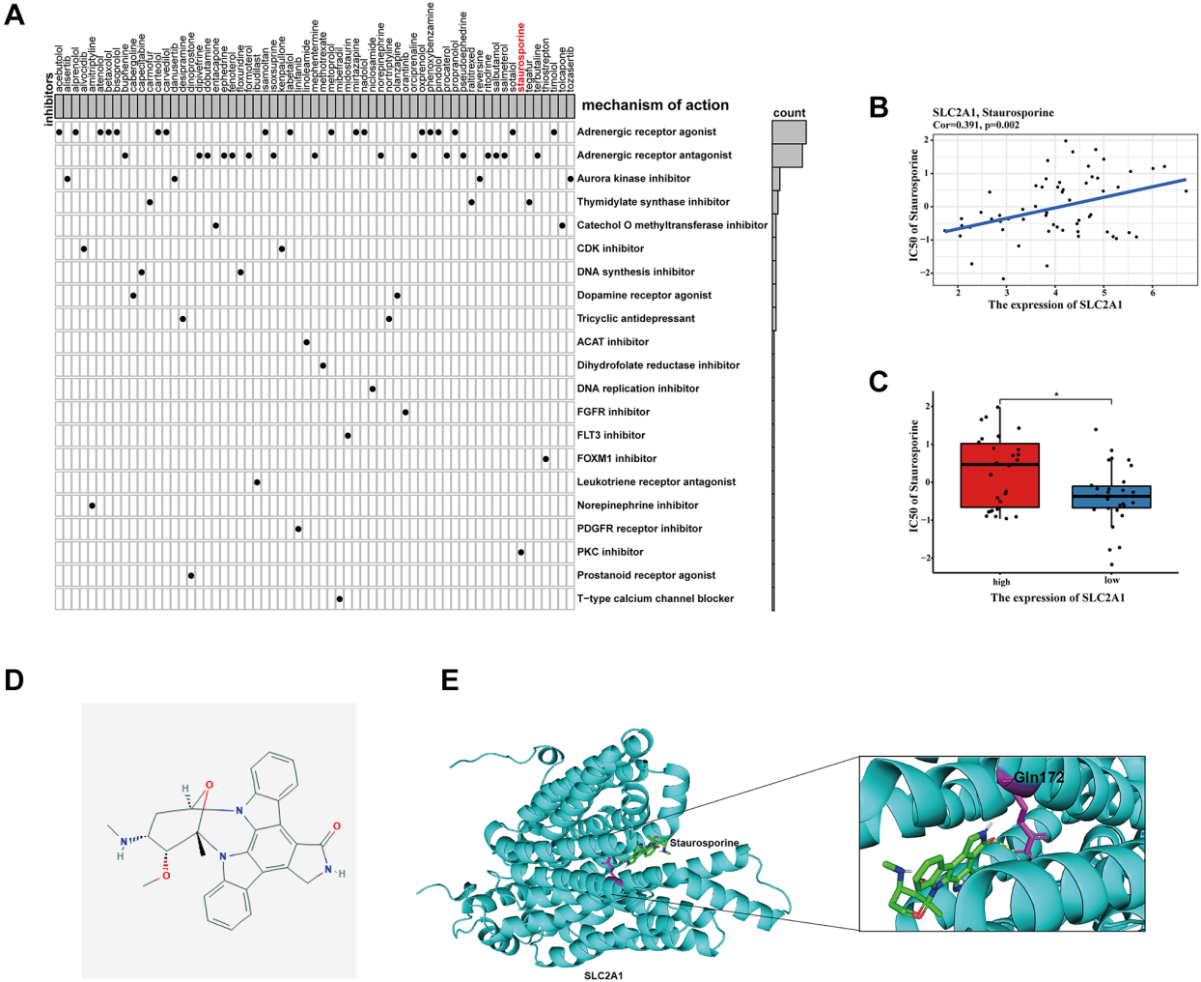
**Identification of candidate drugs targeting the risk signature.** (**A**) Results of CMap analysis. (**B**–**C**) Correlation between SLC2A1 expression and predicted drug response. (**D**) 2D molecular structure diagram of staurosporine. (**E**) The 3D interaction diagrams of Staurosporine and SLC2A1. ^*^P < 0.05; ^**^P < 0.01; ^***^P < 0.001.

Combination chemotherapy has achieved partial efficacy in patients with advanced lung cancer [15]. Therefore, the sensitivity of chemotherapy drugs between the two groups was evaluated. The estimated IC50 values of Bleomycin, Cisplatin, Docetaxel, Gefitinib, Gemcitabine, Paclitaxel, Vinblastine, and Vinorelbine were all significantly lower in patients in high-risk group (Figure 12), which suggested that the signature could be used as a potential predictor of chemotherapy sensitivity. According to CMap database analysis, a total of 62 compounds indicated 21 mechanisms of action were predicted to target 47 prognostic MAGs (Figure 13A). Furthermore, the correlation between SLC2A1 expression and predicted drug (Staurosporine) response was shown in Figure 13B, and high expression of SLC2A1 required more targeted staurosporine (Figure 13C). 2D molecular structure diagram of staurosporine was shown in Figure 13D. Molecular docking studies were used to explore the possibility of interaction between staurosporine and SLC2A1, staurosporine was most likely to function through the combination of GLN-172 of SLC2A1 (Figure 13E).

### Experimental verification

**Figure 14:**
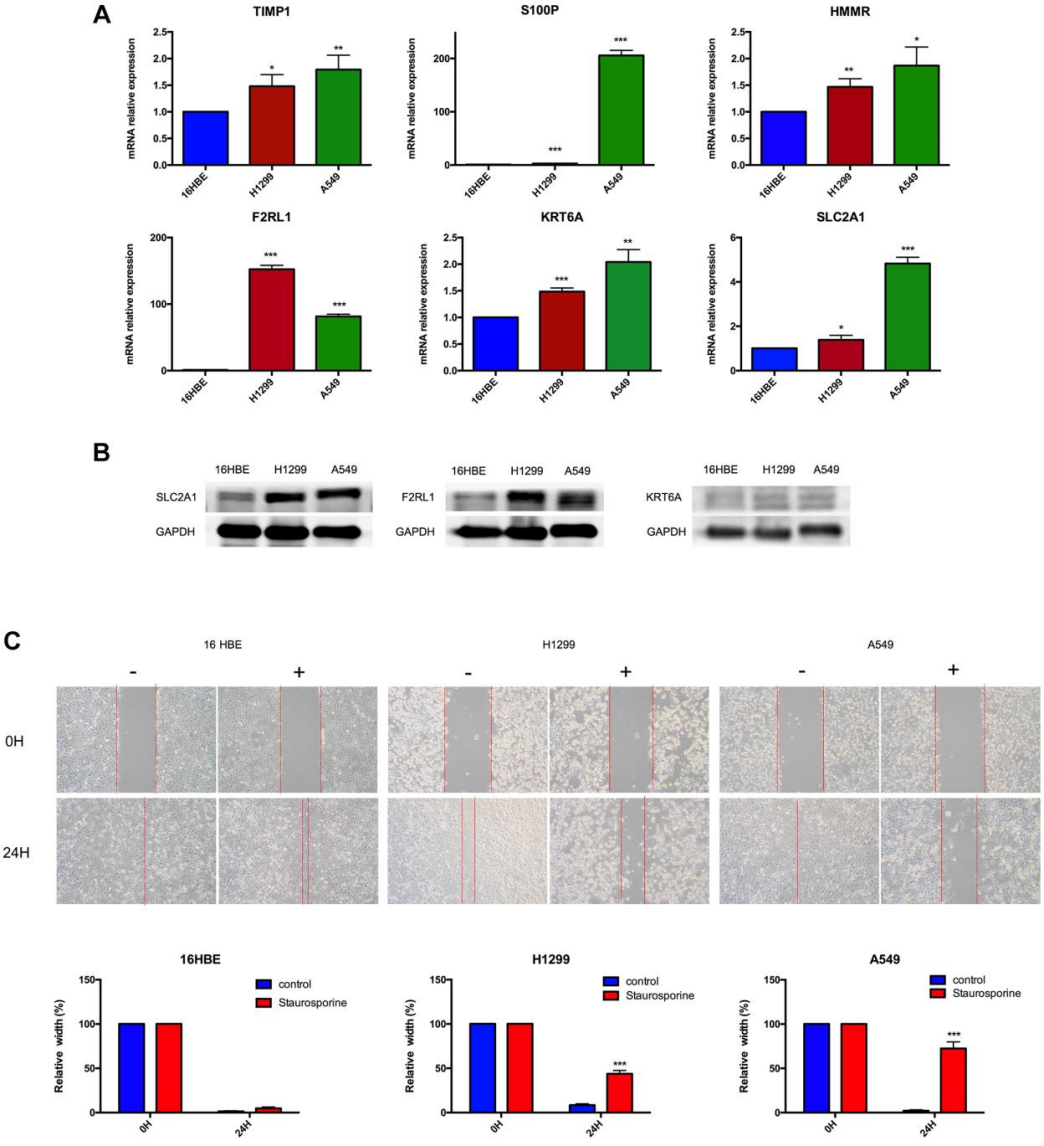
**Experimental verification.** (**A**) Results of TIMP1, S100P, HMMR, F2RL1, KRT6A, and SLC2A1 mRNA expression in A549, H1299 and 16HBE by RT-PCR. (**B**) Results of F2RL1, KRT6A, and SLC2A1 protein expression in A549, H1299 and 16HBE by western blot. (**C**) Staurosporine could inhibit cell migration in A549 and H1299 cells. ^*^p < 0.05, ^**^p < 0.01, ^***^p < 0.001.

In order to further validate 6 MAGs expression in the lab, qRT-PCR in normal respiratory epithelial cells (16HBE) and 2 lung adenocarcinoma cell lines (A549 and H1299) were carried out. As illustrated in Figure 14A, mRNA expression levels of TIMP1, S100P, HMMR, F2RL1, KRT6A, and SLC2A1 were significantly increased in lung adenocarcinoma cell lines compared to16HBE, which were consistent with our bioinformatics analysis results. Besides, the protein expression of SLC2A1, F2RL1 and KRT6A were also significantly increased in A549 and H1299 compare to 16HBE (Figure 14B). Furthermore, we validated effect of SLC2A1 predicted drug (staurosporine) on cell metastasis by migration experiment (Figure 14C), as shown that staurosporine could significantly inhibit A549 and H1299 migration.

## DISCUSSION

Metastasis is the most important reason affecting patient prognosis in LUAD patients, and analyzing transcriptome differences of metastatic tumors will help to identify factors affecting metastasis and predict clinical outcomes. The present study sought to address these gaps in knowledge by comparing transcriptome profiles between metastatic and primary tumors, and 192 genes were identified as metastasis-associated genes. Furthermore, 47 genes associated with the prognosis of LUAD patients were screened. In a prognostic model of MRGs based on Lasso Cox regression analysis, we found a shorter survival time in the high-risk score group. Clinically, patient's age, gender and TNM stage were often used to assess the prognosis. We combined the signature model with clinical risk factors, found that when patients were exposed to the same clinical risk factors, the high risk group had a significantly worse prognosis, which may provide a more accurate prognosis for each patient with advanced lung adenocarcinoma.

The metastasis-associated prognostic signature was constructed basing on six MAGs. Six MRGs have been found to be involved in the progression of tumor metastasis in many tumors. TIMP1 is a member of the tissue inhibitor of metalloproteinase (TIMP) family, which is prominently appreciated as natural inhibitors of cancer-promoting metalloproteinases [16]. TIMP-1 expression was found to correlate positively with cancer progression, such as myeloma [17], non-small cell lung cancer [18] and endometrial carcinoma [19]. This study explored that TIMP-1 mRNA expression was increased in A549 and H1299, and high expression of TIMP-1 was closely related to the metastasis of lung adenocarcinoma. S100 calcium-binding protein P (S100P) is a small calcium-binding protein of S100 family, involving in promoting a number of pathways for proliferation, migration, and invasion [20]. Mingdao Lin et al. reported that S100P contributed to promoter demethylation to promote metastasis in colorectal cancer [21]. Small molecule inhibitors of S100P were found have anti-metastatic effects on pancreatic cancer cells [22]. Hsu YL et al. reported that S100P interacted with integrin α7 and increased cancer cell migration and invasion in lung cancer [23]. In agreement with these findings, S100P mRNA expression was increased in A549 and H1299, the same as result from the HPA database. HMMR (hyaluronan-mediated motility receptor) has been revealed to be associated with reduced overall survival in lung cancer patients [24]. HCG18/miR-34a-5p/HMMR axis were found could accelerate the progression of lung adenocarcinoma [25]. HMMR was found could serve as a novel biomarker associated with progression and prognosis of bladder cancer [26]. High levels of HMMΔexon 8-16 could accelerate pancreatic cancer progression by collaborating with dysfunctional p53 [27]. Also in line with this research, HMMR mRNA expression was increased in A549 and H1299, the same as result from the HPA database, which was significantly negatively associated with the prognosis. F2RL1 was found could be one of thirteen immune-related genes as prognostic signatures in colorectal cancer [28]. We further explored F2RL1 mRNA and protein expression were increased in A549 and H1299. Keratin 6A (KRT6A) is a critical component of cytoskeleton in mammalian cells. KRT6A was reported could promote lung cancer cell growth and invasion [29], it also could serve as invasion-related gene signature predicts prognostic features of LUAD [30]. Consistent with the above studies, we also found that KRT6A could be used as prognostic markers of lung adenocarcinoma metastasis related genes, and on this basis, we further confirmed its mRNA and protein expression were increased in A549 and H1299. SLC2A1 gene encodes GLUT1, which is a glucose transporter that mediates glucose metabolism in cancer cells [31]. SLC2A1 is identification to be differentially expressed genes in non-small cell lung cancer [32]. SLC2A1 can be used as a biomarker for the diagnosis and treatment of esophageal carcinoma [33], pancreatic carcinoma patients [34]. Consistent with the above studies, we not only found that SLC2A1 could be used as a prognostic gene for lung adenocarcinoma metastasis, but confirmed its mRNA and protein expression were increased in A549 and H1299. Furthermore, this study showed that staurosporine could be anchored to SLC2A1 as a targeted drug through GLN-172, its effect on inhibition of cell migration was confirmed in A549 and H1299.

The metastasis-associated signature can not only be used to evaluate the prognosis of patients with advanced LUAD, but also be useful in guiding treatment. Patients with advanced lung adenocarcinoma often have lost the opportunity for surgery, comprehensive treatment methods including chemotherapy, radiotherapy, targeted therapy, and immunotherapy. This study found that the high-risk group have high sensitivity of chemotherapy drugs. A total of 62 compounds indicated 21 mechanisms of action were predicted to target 47 prognostic MAGs. The high-risk group patients showed higher expression of CD274 (PD-L1), PDCD1LG2 (PD-L2), TNFSF4/7/9, TNFRSF9 and IDO1. The high risk group undergoing anti-PD-1/PD-L1 therapy had a better progression-free survival (PFS) than low risk group.

However, we have to admit that there are some limitations to be improved. Firstly, we validated our metastasis-associated prognostic signature in other three independent cohorts (GSE31210, GSE50081, and GSE68465 cohort). There are 164 patients with advanced lung adenocarcinoma in GSE68465 cohort, but GSE31210, GSE50081 do only includes early stage lung cancer, in the future we need collect more patient's information to validate the constructed prognostic signature. Secondly, how those findings can be used as a translational research needs further research. For example, combine clinical cases and patient' s tissue samples to further confirm the validity of this signature. Thirdly, more experiment data to further explore the effect of 6 hub genes on metastasis of advanced lung adenocarcinoma were needed.

In conclusion, this study found six MAGs can be used as a prognostic factor of lung adenocarcinoma metastasis, and constructed a new signature model basing six genes for prognosis of LUAD patients. The high risk group had a significantly worse prognosis. For first time, this study found that the high-risk group have high sensitivity of chemotherapy drugs, higher expression of CD274 (PD-L1), PDCD1LG2 (PD-L2), TNFSF4/7/9, TNFRSF9 and IDO1 and is more effective for PD-L1immunotherapy. Finally, 62 targeted drugs were found, and staurosporine was identified as a targeted drug for SLC2A1, which could inhibit cell migration in A549 and H1299.

## METHODS

### Data sources and processes

Clinical and gene expression data for LUAD samples were available from the GEO (https://www.ncbi.nlm.nih.gov/geo/) and TCGA (https://portal.gdc.cancer.gov/) database. The gene expression datasets (GSE10072 [35], GSE18842 [36], GSE31320 [37], GSE32863 [38], GSE40791 [39], GSE43458 [40], GSE75037 [41] and TCGA-LUAD) were obtained from these databases. A list of metastasis-associated genes (MAGs) were retrieved from the HCMDB database (http://hcmdb.i-sanger.com/). Log2 conversion and normalization were conducted for the expression profiles of each dataset. The “ComBat” algorithm in R package sva was employed to remove batch effects.

### Identification and enrichment analysis of differentially expressed MAGs

To investigate differentially expressed MAGs among each GEO dataset, |logFC|>1 and corrected *P* < 0.05 was considered to be significant by using R package limma. RobustRankAggreg (RRA) method was used to obtain robust MAGs that were ranked consistently better than expected by chance with the R package RRA [42]. To further identify function of these genes, GO and KEGG enrichment analyses were performed with R package clusterProfiler for exploring biological process, molecular function and pathway [43].

### Construction and validation of metastasis-associated prognostic signature

TCGA cohort samples were classified as the training cohort and three GEO cohort samples were classified as the test cohort. Firstly, univariate Cox regression analysis was conducted to find survival-related MAGs. Next, Lasso and multiple Cox regression analyses were performed to screen prognostic MAGs for constructing the prognostic model. The risk score of LUAD patients was calculated as the following formula:


$$\text{Risk score}=\sum_{\text{g}=1}^{\text{n}}\text{coef}(\text{g})\times \text{x}(\text{g})$$

where coef (g) was the coefficient of candidate MAGs and x(g) was the standardized expression levels of each MAGs, respectively. On the basis of the median risk score, patients were classified into the high and low risk groups. Univariate and multivariate Cox regression analyses were used to identify independent prognostic factors affecting the prognosis of LUAD patients. Kaplan-Meier (K-M) survival analysis and time-dependent receptor operating characteristic (ROC) curve was utilized to assess the predictive performance of the metastasis-associated prognostic signature [44]. Finally, the reliability and applicability of the prognostic signature was further validated in the GEO cohorts (GSE31210, GSE50081 [45] and GSE68465 cohort). For providing an intuitive visualization of the prognostic signature, the nomogram was constructed using R package rms. Meanwhile, calibration curves were generated to estimate the accuracy of the nomogram. The hub genes protein expression level was determined using HPA database (https://www.proteinatlas.org/).

### Correlation between the risk score and immune microenvironment characteristics

TIMER [46], CIBERSORT [47], QUANTISEQ [48], MCPcounter [49], xCELL [50] and EPIC [51] algorithms were applied to evaluate the relative abundance of infiltrating immune cell subsets among high- and low-risk groups. To investigate the activity of immune cells and immune-related pathways of each LUAD sample, Single sample gene set enrichment analysis (ssGSEA) was then employed using R package GSVA [44]. Immune checkpoint blockade key genes were collected from previous research.

To understand whether the signature was more effective for immunotherapy, the predictive value of the signature was assessed on the GSE135222 [52] and GSE126044 [53] datasets treated with immunotherapy. In addition, The Tumor Immune Dysfunction and Exclusion (TIDE) (http://tide.Dfci.harvard.edu/) was leveraged to evaluate the potential clinical efficacy of immunotherapy for the signature based on the gene expression profile of TCGA-LUAD samples. Lower TIDE scores indicated a lower potential for immune evasion, suggesting that patients were more likely to benefit from immunotherapy [54].

### Exploration of the significance of the signature in the clinical treatment

To evaluate the prognostic signature in the clinic for LUAD patient treatment, we calculated the half maximal inhibitory concentration (IC50) of common administrating chemotherapeutic drugs. The IC50 values of drugs in cancer cell lines were downloaded from the Genomics of Drug Sensitivity in Cancer (GDSC) database [55]. According to the GDSC database, the chemotherapy response for Bleomycin, Cisplatin, Docetaxel, Gefitinib, Gemcitabine, Paclitaxel, Vinblastine, and Vinorelbine of each LUAD patient was implemented using R package pRRophetic [55].

### Identification of candidate drugs

To present potential candidate drugs, the CMap database was conducted to predict small molecule drugs on the base of the prognostic MAGs as previously described [9]. The correlation between hub MAGs expression and drug response was predicted by CellMiner database (https://discover.nci.nih.gov/cellminer/) [56]. The structure of staurosporine and SLC2A1 were acquired from PubChem Compound (https://www.ncbi.nlm.nih.gov/pccompound, PubChem CID: 44259) and AlphaFold Protein Structure Database (https://www.alphafold.ebi.ac.uk/entry/P11166), respectively [57]. The molecular docking was conducted via AutoDockTools 1.5.6, Vina and Genetic Algorithm, and the docking result was displayed by PyMoL.

### Statistical analyses

Data Analyses were conducted in the same way as in the previous article [9].
